# Design and Evaluation of a *Cinnamomum burmannii* Essential Oil-Loaded Preservative Film for Enhancing the Quality and Shelf Life of *Squaliobarbus curriculus* Filets

**DOI:** 10.3390/foods14173139

**Published:** 2025-09-08

**Authors:** Xiaonan Zhang, Jiayi Lai, Xiaoxiao Dai, Feng Huang, Lei Guan, Rushu Wen

**Affiliations:** 1 Life Science College, Jiaying University, Meizhou 514015, China; 18316404107@163.com (J.L.); daixiaoxiao0201@163.com (X.D.);; 2 Aquatic Research Institute, Meizhou Academy of Agricultural and Forestry Sciences, Meizhou 514015, China

**Keywords:** essential oil, *Cinnamomum burmannii*, *Squaliobarbus curriculus*, bacteriostatic activity, biodegradable food packaging

## Abstract

In this study, an edible matrix consisting of sodium alginate, gelatin, zein, and gum arabic was combined with *Cinnamomum burmannii* essential oil (CBEO) to produce a natural, eco-friendly, and bioactive food packaging preservation film. After the CBEO was extracted by hydrodistillation and analyzed using gas chromatography mass spectrometry, 55 chemicals were found, with the main ingredients being α-terpineol, borneol, and cinnamon aldehyde. Scanning electron microscopy and Fourier transform infrared spectroscopy were used to extensively evaluate the preservative coating, which demonstrated bacteriostatic activity. When compared to the control at a 3% CBEO loading, the film effectively maintained color stability while extending the shelf life of *Squaliobarbus curriculus* filets by around 3 times. Furthermore, compared to the blank film, the film showed a 23.8% increase in tensile strength and a 23.59% improvement in light transmittance. These results show how CBEO-loaded edible films can enhance meat preservation and offer fresh perspectives on the creation of useful, biodegradable food packaging materials.

## 1. Introduction

*Squaliobarbus curriculus*, a representative fish of the Hakka region in southern China and a key ingredient of the traditional delicacy “Yusheng” [1,2], is rich in high-quality proteins and polyunsaturated fatty acids [3]. However, its extremely short shelf-life poses a significant challenge, as quality deterioration—including discoloration, flavor loss, and softening—begins within hours post-slaughter, and refrigeration at 4 °C extends it only up to 48 h [4]. Conventional preservation techniques, such as ice storage or freezing, can slow spoilage [5,6], but freezing induces ice crystal formation that damages cellular structures and reduces texture and tenderness [1,7,8,9,10]. The occasional use of chemical preservatives raises consumer safety concerns, while widely applied nondegradable plastic packaging imposes a substantial environmental burden [11,12,13,14,15]. These limitations highlight the urgent need for effective, safe, and sustainable preservation strategies, motivating the development of bioactive preservative films in this study.

Natural biopolymer-based preservative films have drawn interest in food preservation in recent years due to their safety, biodegradability, and environmental friendliness [16,17,18]. These films were made of chitosan and included thyme essential oil, which successfully prolonged the shelf-life of Oncorhynchus mykiss filets.

Similarly, Ref. [19] used cinnamon essential oil and sodium alginate to prepare coatings that suppressed *Escherichia coli* and *Staphylococcus aureus* while significantly prolonging the shelf-life of perch filets [20]. demonstrated that gum arabic-stabilized zein nanoparticles could efficiently encapsulate peppermint oil, with nanoemulsions prepared Via the propylene glycol solvent displacement method exhibiting good sustained-release properties, achieving 100% release within 24 h under acidic conditions. Additionally, the active ingredients of essential oils are protected by natural polymer materials like gelatin and chitosan, which also improve the physicochemical stability of film matrices [21,22,23]. This allows for targeted release in areas contaminated by microorganisms, which lowers the dosage needed [24].

*Cinnamomum burmannii* is an evergreen tree of the Lauraceae family, widely distributed in Southeast Asia and southern China. Its leaves are rich in essential oils, whose major components include oxygenated monoterpenes (e.g., borneol, α-terpineol) and aldehydes (e.g., cinnamaldehyde). Previous studies have demonstrated that *C. burmannii* essential oil exhibits strong antimicrobial activity against foodborne pathogens [25] and possesses antioxidant properties through free radical scavenging [26], making it a promising natural preservative for the food industry. However, its application in freshwater fish preservation, particularly for *Squaliobarbus curriculus*, remains largely unexplored.

This study aimed to develop a natural preservative film by incorporating *Cinnamomum burmannii* essential oil (CBEO) into a zein–gum arabic nanoparticle system and blending it with a sodium alginate-gelatin matrix. To our knowledge, this is the first application of CBEO in preserving *Squaliobarbus curriculus* filets. The objectives were to optimize the film formulation for improved stability and functionality, evaluate its physicochemical and antimicrobial properties, and assess its potential as a sustainable alternative to synthetic preservatives and non-degradable packaging.

## 2. Materials and Methods

### 2.1. Materials and Chemicals

*Cinnamomum burmannii* leaves were collected from the campus of Jiaying University in Meizhou city, China. Select pest-free, intact, and mature leaves, wash thoroughly, and place in a well-ventilated area at room temperature (25 ± 2 °C) for 15 min to air dry before use. Fresh *Squaliobarbus curriculus* samples were purchased from the Meizhou Agricultural Product Market, with undamaged and physiologically sound individuals chosen for experimentation. The samples were first stunned with a wooden mallet, followed by careful evisceration to remove the viscera and gills. Filets were trimmed into uniform triangular pieces (25 mm × 25 mm × 50 mm, approximately 5 mm thick) to ensure dimensional consistency across samples. The following raw materials and reagents were used in the experiment: Sodium alginate (Tianjin Zhonglian Chemical Reagent Co., Ltd., Tianjin, China): purity ≥ 95%, viscosity ≤ 180 ± 20 mPa·s, water-insoluble substances ≤ 0.1%; Gum arabic (Tianjin Huasheng Chemical Reagent Co., Ltd., Tianjin, China): purity ≥ 99.0%, viscosity 80–120 mPa·s, pH 4.1–4.6, moisture content ≤ 15%, ash content ≤ 3.5%; Zein (Shanghai Yuanye Bio-Technology Co., Ltd., Shanghai, China): bioreagent grade, protein content ≥ 92%; Gelatin (Guangdong Kangda Biotechnology Co., Ltd., Guangzhou, China), food grade.

### 2.2. Instrument

Laboratory pure water system, model: Master-Touch-S30UVF (Shanghai, China); constant temperature and humidity incubator, model: GZX-150BSH/QHX (Shanghai, China); UV-Vis spectrophotometer, model: T-600A (Beijing, China); electronic nose sensor, model: PEN3 (Schwerin, Germany); high-pressure homogenizer, model: HPH-L2 (Suzhou, Jiangsu, China); magnetic stirrer, model: C-MAGHS7 (Staufen, Germany); electronic balance, model: SQP/QUINTIX51 (Göttingen, Germany).

### 2.3. Fabrication of Active Preservative Films

#### 2.3.1. Preparation of Essential Oil Nanoemulsion

The preparation of the essential oil nanoemulsion followed the method outlined by [27], with minor modifications. The process involves 3 steps: (1) Preparation of zein solution: 1 g of zein was added to 25 mL of ethanol aqueous solution (70%, *v*/*v*) and stirred for 2 h to obtain a homogeneous zein solution. Separately, 50 mL of zein/deionized water solution was prepared at a 1:3 ratio (zein: water) under constant stirring for 30 min. (2) Gum arabic solution preparation: To guarantee full dissolution, 50 mL of gum arabic/deionized water solution was prepared at a 1% (*w*/*v*) concentration and agitated at 450 rpm for 40 min. (3) Synthesis of nanoemulsion: In total, 49 mL of gum arabic solution was gradually mixed with 1 mL of *Cinnamomum burmannii* essential oil while being constantly stirred. The mixture was then homogenized using a high-pressure homogenizer (HPH-L2, Nantong, China) at 50 MPa for five cycles to form a stable nanoemulsion. Subsequently, 50 mL of zein solution was gradually added to the nanoemulsion containing different essential oil concentrations, yielding zein-gum arabic nanoemulsions loaded with 1%, 2%, and 3% *Cinnamomum burmannii* essential oil, respectively.

#### 2.3.2. Preparation of Preservative Films

With a few minor adjustments, the preservative film preparation process was based on the methodology described by [28]. Firstly, 2% (*w*/*w*) sodium alginate solution (100 mL) was made by dissolving sodium alginate in deionized water after it had been weighed. For 45 min at room temperature, the mixture was magnetically agitated at 500 rpm until it completely dissolved and became a homogenous mixture. Gelatin was dissolved separately in deionized water to create a 10% (*w*/*w*) gelatin solution (100 mL), which was then continuously stirred in a water bath at 60 °C until it completely dissolved. After that, 4 mL of glycerol was added as a plasticizer to the mixture of sodium alginate and gelatin solutions at a mass ratio of 6:4 (sodium alginate–gelatin). At 40 °C and 600 rpm, the mixture was agitated until it was evenly combined. Then, at a volume ratio of 1:1 (nanoemulsion to matrix solution), *Cinnamomum burmannii* essential oil nanoemulsions at different concentrations (1%, 2%, or 3%), were added to the film-forming solution and well mixed to guarantee homogeneity. All films were dried under controlled conditions at 60 °C and relative humidity of 50 ± 5%. Maintaining consistent temperature and humidity during drying is critical for ensuring uniform film formation, preventing defects such as cracking or uneven thickness, and achieving reproducible mechanical and optical properties.

### 2.4. Scanning Electron Microscopy Observation of Preservative Films

Using the methodology outlined by [29], scanning electron microscopy (SEM, model: Hitachi SU5000, Tokyo, Japan) was used for sample observation and analysis in order to examine the surface morphology and microstructure of the *Cinnamomum burmannii* essential oil composite preservative film. Double-sided conductive copper tape was used to mount the prepared film samples onto a gold-plated sample table after they were sliced into tiny pieces (5 mm × 5 mm). The samples were sputter-coated with a nanoscale gold coating to improve the conductivity and imaging clarity. Imaging was carried out using a working distance of 10 mm and an accelerating voltage of 20 kV. Each film formulation was analyzed in triplicate, with representative images selected for comparative analysis to elucidate the impact of varying essential oil concentrations on the microstructure of the film.

The physicochemical properties of the nanoemulsions were characterized using dynamic light scattering (DLS, Zetasizer Nano ZS90, Malvern Instruments, Malvern, UK) to evaluate their stability and uniformity. Each formulation (1%, 2%, and 3% essential oil) was measured in triplicate at 25 °C. The results indicated that the nanoemulsion loaded with 3% essential oil exhibited the optimal performance, with an average particle size of 178.5 ± 9.6 nm, a polydispersity index (PDI) of 0.20 ± 0.04, and a zeta potential of −34.8 ± 2.5 mV. These parameters indicate a narrow particle size distribution (PDI < 0.3) and strong electrostatic stability (absolute zeta potential > 30 mV), which are crucial for preventing droplet aggregation during storage and ensuring uniform incorporation into the film matrix. The 1% and 2% essential oil-loaded nanoemulsions showed similar trends, with average particle sizes of 189.2 ± 8.8 nm and 172.6 ± 8.3 nm, PDIs of 0.23 ± 0.05 and 0.19 ± 0.03, and zeta potentials of −32.5 ± 2.1 mV and −35.7 ± 2.2 mV, respectively.

### 2.5. Fourier Transform Infrared Spectroscopy Observations of Preservative Films

Fourier transform infrared spectroscopy (FTIR, model: Nicolet Magna-IR560, Madison, WI, USA) was performed according to the method described by [30]. The dried film samples were cut into appropriate sizes, placed on KBr pellets, and heated under an infrared lamp to soften and melt. Another KBr pellet was then placed on top, and the assembly was pressed under controlled pressure to form a thin, homogenous film. Spectral data were acquired over a wavenumber range of 500–5000 cm^−1^ with a resolution of 4 cm^−1^ and 32 scans per sample to increase the signal-to-noise ratio. Characteristic absorption peaks corresponding to functional groups in the film matrix were analyzed to compare the effects of varying essential oil concentrations on molecular interactions.

### 2.6. Measurement of the Light Transmittance of Preservative Films via a UV-Vis Spectrophotometer

The light transmittance of the preservative films was assessed Via a UV-Vis spectrophotometer (Model: UV-5100, Shanghai Yuanxi Instrument Co., Ltd., Shanghai, China) to evaluate their visible light barrier properties. To ensure there were no wrinkles or air bubbles, dry film samples were cut into rectangular strips of the proper size and adhered smoothly to one side of a colorimetric cuvette in accordance with conventional protocols. The reference was a cuvette that was blank-that is, devoid of film. A wavelength of 700 nm was used to test each film sample’s absorbance [31] (*A*). The average absorbance value was determined after each sample was examined in triplicate. The following formula was then used to calculate the light transmittance (*T*):

(1) $$T\;(\%)=10^{2-A}$$

where *T* is the light transmittance (%) of the film and *A* is the absorbance at 700 nm.

### 2.7. Analysis of Volatile Odor Compounds via Electronic Nose

The PEN3 electronic nose (AIRSENSE GmbH, Schwerin, Germany) was used to assess the fish samples’ volatile odor profiles in accordance with the technique outlined by [32]. Ten different metal oxide gas sensors are included with the PEN3 system; these sensors have different sensitivities: W1C (aromatic compounds), W5S (nitrogen oxides), W3C (aromatic compounds), W6S (sulfides/reducing gases), W5C (alkanes/aromatic compounds), W1S (methane derivatives), W1W (sulfur-containing compounds), W2S (alcohols/ketones), W2W (aromatic/organosulfur compounds), and W3S (alkanes/aliphatic compounds). The fish samples (2 g) were put into 50 mL sealed headspace vials and let to equilibrate for half an hour at room temperature in order to help the volatile chemicals come out. The e-nose’s dual-needle sampling probe was placed into each vial before the measurement. The following operational parameters were used throughout the analysis: 400 mL/min of carrier gas flow rate, 5 s wait time, 360 s purge time, and 1 min measurement time.

Sensor responses were recorded as the ratio of the sample resistance to the reference air resistance (G/G_0_), providing a quantitative representation of odor characteristics. The collected data were subjected to multivariate statistical analyses, including principal component analysis (PCA) and hierarchical clustering, Via Origin 2022 software to assess odor profile differences among treatment groups and evaluate the effectiveness of preservation strategies.

### 2.8. Detection of Volatile Compounds in Samples by Gas Chromatography–Mass Spectrometry

Following the procedure reported by [30], the extracted essential oil was first dehydrated with anhydrous sodium sulfate (1:5, *w*/*w*) and left to stand for 2 h to remove residual moisture. The sample was then filtered through a 0.22 μm organic-phase membrane to obtain a clear oil. The volatile constituents of the purified essential oil were subsequently analyzed by gas chromatography–mass spectrometry (GC–MS) using an Agilent 7890 system. Separation was achieved on a DB-1 capillary column (60 m × 0.25 mm × 0.25 μm). The oven temperature program was as follows: initial temperature of 40 °C (held for 1 min), ramped at 5 °C/min to 150 °C, then at 10 °C/min to 200 °C. High-purity nitrogen (N_2_) was used as the carrier gas at a constant flow rate of 1.0 mL/min. GC–MS analysis conditions were applied as described in [33], which provides a validated method for essential oil volatile profiling.

### 2.9. Physical Properties of the Preservative Film

Tensile testing was performed Via an HLD tensile tester (Aidebao Instrument Co., Ltd., Yueqing, China). The preservative film samples were cut into rectangular strips (15 mm × 40 mm), mounted onto the grips, and subjected to a constant-speed tensile force until rupture. The maximum tensile force (*F_max_*) and displacement at break were recorded. Each group of preservative films was tested in triplicate, and the average values were calculated. Following the formula from [34], the tensile strength (TS) and elongation at break (EB) were calculated as follows:

(2) $$\mathrm{TS}\;\left(\mathrm{MPa}\right)=\frac{F_{max}}{S}$$

where *F_max_* is the maximum tensile force at preservation film rupture, and *S* represents the cross-sectional area of the preservative film.

(3) $$EB\;\left(\%\right)=\frac{L_{max}-L_{0}}{L_{0}}\times 100\%$$

*L_max_* is the length of the preservative film at rupture, and *L*_0_ represents the original length of the preservative film. The thickness of the film samples was measured at six randomly selected points Via a handheld micrometer (Mitutoyo Corp., Kanagawa, Japan; measurement accuracy: 0.01 mm), and the average value was calculated.

### 2.10. Inhibitory Activity Experiments of Escherichia coli and Staphylococcus aureus

The inhibitory effects of *Cinnamomum burmannii* essential oil on *Escherichia coli* and *Staphylococcus aureus* were evaluated Via a modified agar disk diffusion method [35]. Sterile filter paper disks were impregnated with *Cinnamomum burmannii* essential oil or 4 μg/mL ampicillin solution. In a Petri dish (60 mm × 60 mm), 10 mL of sterilized LB agar medium was poured evenly and allowed to solidify. Subsequently, 100 μL of bacterial suspension was uniformly spread on the medium surface. The impregnated disks were then placed at the center of the agar plate and incubated at 37 °C for 16 h. The diameter of the inhibition zones was measured, and the inhibition rate (%) was calculated as follows:

(4) $$Inhibition\;ratio\;\left(\%\right)=\frac{D_{i}}{D}\times 100$$

where *D_i_* represents the inhibition zone diameter (mm) and *D* represents the diameter of the bacterial inoculation area (mm).

### 2.11. Changes in the Physicochemical Quality and Color Attributes of Squaliobarbus curriculus Filets Treated with Preservative Film and Microbiological Analysis

Untreated fish filets served as the negative control, whereas filets treated with potassium sorbate solution were designated the positive control. *Squaliobarbus curriculus* filets were preserved using films containing 1%, 2%, or 3% *Cinnamomum burmannii* essential oil. The experiment was conducted under controlled conditions (Temperature: 29.0 ± 0.5 °C; relative humidity: 75%). The physical and chemical indicators of the fish meat were measured every 24 h, with photographic documentation of the experimental process and outcomes. Following the methods of [36], fish slices were placed in polystyrene Petri dishes (90 mm diameter), and color changes were quantified Via a colorimeter (ZE-6000, Tokyo, Japan) at designated storage intervals. The color parameters brightness (L*), redness (a*), and yellowness (b*) were statistically analyzed to evaluate the impact of different treatments on the visual quality of the fish meat.

The changes in total viable count (TVC) during storage were determined using the pour plate method on Plate Count Agar (PCA), following the procedures described by [37] with slight modifications. Briefly, 10 g of fish sample from each treatment group was aseptically weighed and homogenized with 90 mL of sterile saline solution (0.85% NaCl) in a stomacher bag for 2 min. Subsequent serial decimal dilutions were prepared. Then, 1 mL of appropriate dilutions was poured onto Plate Count Agar (PCA, Hope Bio-Technology Co., Ltd., Qingdao, China) and incubated at 37 °C for 48 h. After incubation, colonies were counted and expressed as logarithm of colony-forming units per gram of sample (log10 CFU/g). The analysis was performed in triplicate for each sample at each sampling point.

### 2.12. Statistical Analysis

All experiments, including the measurement of physical properties of preservative films and the quantification of essential oil components by GC–MS, were performed in at least 3 independent replicates. Data are expressed as mean ± standard deviation (SD). Statistical differences among films containing different concentrations of *Cinnamomum burmannii* essential oil (EO) were analyzed using one-way analysis of variance (ANOVA), followed by Tukey’s post hoc test for multiple comparisons. For GC–MS component distributions, relative peak areas were compared across samples using the same ANOVA and post hoc approach. A *p*-value < 0.05 was considered statistically significant. All statistical analyses were performed using GraphPad Prism 9.5.1 (GraphPad Software, San Diego, CA, USA). Figures and plots, including the lollipop plot for GC–MS data, were generated using Origin 2023 (OriginLab, Northampton, MA, USA).

## 3. Results and Discussion

### 3.1. Microscopic Morphology of the Preservative Films

**Figure 1:**
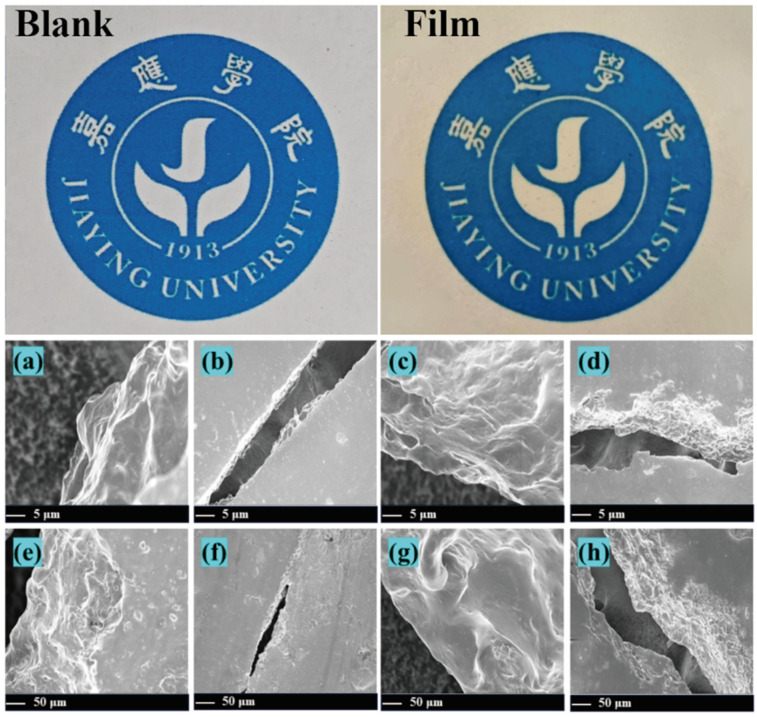
Visual appearance of fish filets under different treatments at selected time points. (**a**,**e**) Blank control; (**b**,**f**) preservative film containing 1% essential oil; (**c**,**g**) preservative film containing 2% essential oil; (**d**,**h**) preservative film containing 3% essential oil.

As illustrated in Figure 1, the blank control sample exhibited a continuous, compact, and irregular surface structure. Sodium alginate and gelatin matrices formed a homogeneous network through intermolecular hydrogen bonding. Observations revealed that zein–gum arabic nanoparticles adopted a spherical morphology and were uniformly embedded within the matrix without detectable phase separation. Upon essential oil incorporation, well-defined nanoencapsules with sharp boundaries were uniformly distributed in the matrix. This suggests that the hydrophobic core of zein encapsulates essential oil molecules, forming a typical core–shell configuration. The carboxyl groups in gum arabic and the hydroxyl groups in sodium alginate synergistically stabilized the microcapsule interfaces via hydrogen bonding and hydrophobic interactions, ensuring the structural integrity of the preservative film. With increasing essential oil concentration, the preservative film structure maintained a uniform dispersion, whereas the surface gradually developed a dense, honeycomb-like microcavity architecture. In our study, although the film thickness increased slightly (from 0.17 mm in the blank control to 0.23 mm in the film containing 3% essential oil), the measured transmittance also increased (from 32.28% to 35.97%), this seemingly contradictory behavior can be explained by structural homogenization induced by high-pressure homogenization of the essential oil–alginate–gelatin emulsion system. Specifically, the incorporation of essential oil induced two key effects: (1) swelling interactions between the oil and the polymer matrix (alginate, gelatin, zein, and gum arabic) expanded intermolecular spacing, while zein–gum arabic nanoparticles carrying the oil were uniformly dispersed, filling voids and slightly increasing film thickness; (2) more importantly, the homogenization process transformed the emulsion into a uniform nanocrystalline structure, minimizing local defects such as aggregates or uneven polymer distribution. This structural regularity reduces light scattering, allowing more light to pass through the film despite the increased thickness. The observed slight yellowish tint reflects the natural color of the essential oil but does not substantially affect the overall transmittance measurement.

These pores originated from residual voids left after essential oil volatilization. It is hypothesized that this honeycomb microstructure endows the film with exceptional loading capacity and sustained-release properties, facilitating the controlled release of active components. This structural feature further supports prolonged bacteriostatic activity and antioxidant efficacy, thereby enhancing preservation performance.

### 3.2. Chemical Structure Analysis of Preservative Films via Fourier Transform Infrared Spectroscopy (FTIR)

**Figure 2:**
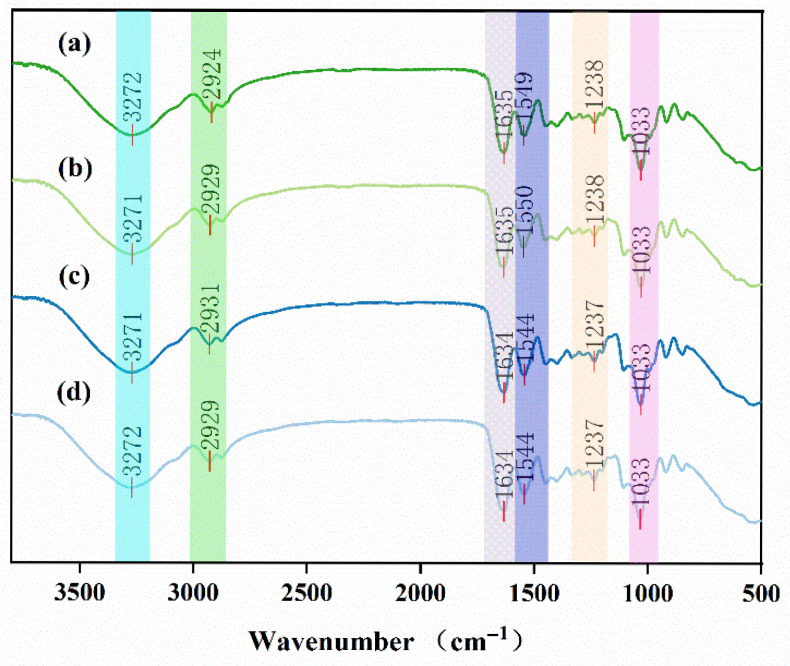
Fourier transform infrared spectroscopy (FTIR) spectra of preservative films with different formulations, highlighting their chemical structural features. (**a**) Film with 3% essential oil. (**b**) Film with 2% essential oil. (**c**) Film with 1% essential oil. (**d**) Blank control film.

Figure 2 presents the FTIR spectra of different preservative film samples used to characterize their chemical structures, the spectra show similar trends across all samples, although minor variations are observed at specific characteristic peaks. The absorption peak at 3271 cm^−1^ is attributed to the stretching vibration of hydroxyl groups (O-H) [38]. The peak at 2924 cm^−1^ corresponds to the asymmetric stretching vibration of CH_2_ groups, which are commonly present in organic compounds, including essential oil constituents, zein, alginate, gelatin, glycerol, and gum arabic [39]. In the present study, this peak was notably more intense in the essential oil-treated films compared to the blank control, suggesting a stronger contribution from terpenes or long-chain alkanes with increasing essential oil content. A distinct peak near 1635 cm^−1^ was observed in all samples, including the blank control. This band is mainly attributed to the stretching vibration of carbonyl groups (C=O) from the carboxylate moieties of alginate, as reported in previous studies on alginate-based films [40]. In the essential oil-loaded films, slight shifts and peak broadening were detected, which may also reflect contributions from aldehydes or ketones of the oil [41] and suggest intermolecular interactions between the oil constituents and the polymeric matrix.

These spectral variations confirm the successful incorporation of cinnamon essential oil into the polymer network. In contrast, the blank film exhibited a comparatively smooth region around 2250 cm^−1^, while the essential oil-containing films showed additional vibrational signals related to the active compounds of the oil. Collectively, the observed peak differences demonstrate the integration of essential oil components into the preservative coatings, despite the overall similarity of the polymeric backbone structures.

### 3.3. Physical Properties of the Preservative Films

The thickness of the blank control film was 0.17 mm, as shown in Table 1. The film thickness rose to 0.20 mm when 1% essential oil was added, to 0.22 mm when 2% essential oil was added, and to 0.23 mm when 3% essential oil was added. The swelling effect between the essential oil and the film matrix, where essential oil molecules increase intermolecular spacing, is probably what caused this rise in thickness. The essential oil-loaded zein–gum arabic nanoparticles may be evenly distributed throughout the matrix, filling in the gaps and adding to the film’s thickness. An HLD digital force gauge was used to measure tensile strength (TS), a crucial parameter for assessing mechanical performance. With a TS value of 6.66 MPa, the blank control film demonstrated good flexibility but a restricted tensile capacity. The TS value increased to 9.73 MPa at 1% EO, suggesting enhanced polymer chain interactions via hydrophobic or hydrogen bonding between essential oil molecules and the film matrix. However, at 2% EO, TS slightly declined to 9.15 MPa, likely due to increased aggregation of zein-gum arabic nanoparticles at higher concentrations, creating stress concentrations that compromise tensile integrity [42]. At 3% EO, TS rebounded to 9.50 MPa, possibly attributed to more complex interfacial interactions (e.g., intensified hydrogen bonding) between nanoparticles, EO, and the matrix, partially offsetting the aggregation effect.

The TS value rose in proportion to the rise in essential oil content. This implies that essential oil molecules might engage in hydrophobic or hydrogen bonding interactions with the film matrix, strengthening the bonds between polymer chains and boosting tensile strength. Additionally, the films’ elongation at break increased significantly, indicating increased flexibility. This could be explained by the active ingredients in the essential oil, like phenolics and terpenoids, which can interact with polar groups in the film matrix through noncovalent means (like hydrogen bonds and hydrophobic forces) to strengthen the network structure and increase the tensile strength and extensibility. Similar findings were reported by [43], who reported that the addition of clove essential oil to chitosan films enhanced both TS and elongation at break at optimal concentrations. Regarding light transmittance, there were no significant fluctuations among the samples. The blank control exhibited a light transmittance of 32.28%, whereas the films with 1%, 2%, and 3% essential oils presented slightly higher values of 33.91%, 34.59%, and 35.97%, respectively. This increase might be due to the formation of an essential oil–alginate–gelatin emulsion, which, after homogenization, produced uniform nanocrystalline structures, increasing the scattering and refraction of light through the film matrix.

### 3.4. The Electronic Nose Determines the Odor of a Sample

#### 3.4.1. Odor Characteristics of Fish Flesh

**Figure 3:**
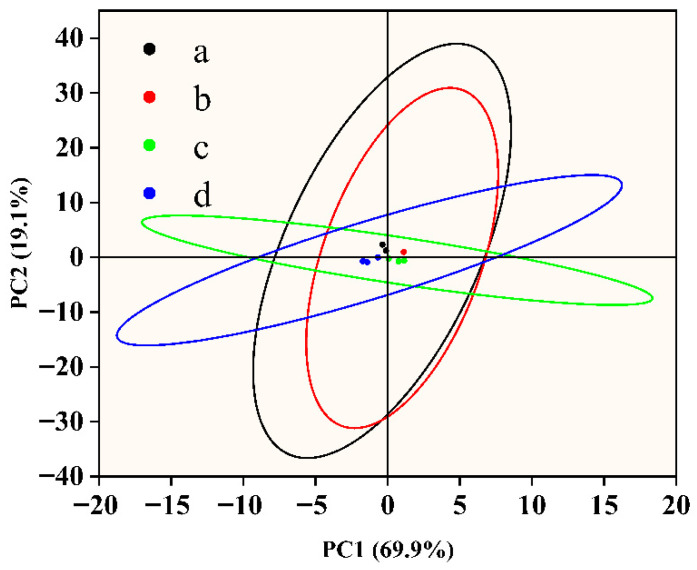
Volatile flavor profiles of fish filets at different storage periods as detected by an electronic nose.

As intelligent analytical instruments that simulate the human olfactory system, electronic noses can detect and identify volatile organic compounds (VOCs) released by fish samples in real time through multiple gas sensors and perform quantitative and classification analyses of their odor characteristics. Figure 3 shows the principal component analysis results of the electronic nose data of the fish samples in the different groups. PC1 and PC2 represent more than 70% of the total variance, and PC2 alone contributes 19.1%, indicating that these two principal components effectively capture the major volatility differences between samples. Further analysis revealed that PC1 was associated mainly with sulfur-containing compounds (such as hydrogen sulfide) and aldehydes (such as hexanal), whereas PC2 was strongly influenced by alcohols (such as ethanol) and ketones (such as acetone), which are common metabolic markers during the deterioration of fish samples. Among them, microbial growth and metabolism are often accompanied by the production of sulfur-containing compounds, whereas lipid oxidation often leads to the accumulation of aldehydes and ketones [44]. In the negative quadrants of PC1 and PC2, the cling film treatment group with additional essential oil displayed tight clustering, suggesting a low volatility and stable volatile component composition. This could be as a result of the fact that nanoencapsulation techniques improve the stability of essential oils, delay their volatilization, and successfully prevent the release of volatile metamorphic chemicals linked to oxidation reactions and microbial metabolism [45]. The untreated group’s samples, on the other hand, were more widely distributed in the PC2 direction, suggesting notable alterations in their volatile properties that might be connected to oxidative rancidity and bacterial fermentation during storage [46,47]. The distinct separation in the PCA plot is corroborated by the GC-MS identification of key spoilage volatiles. Sensors with high loadings on PC1 (W1W, W5S) are likely responding to sulfur-containing compounds and nitrogen oxides, which align with microbial metabolites. Conversely, sensors on PC2 (W2S, W5C) detected alcohols and aldehydes (e.g., Hexanal), consistent with lipid oxidation products identified by GC-MS. This confirms that the E-nose effectively captured the specific volatile fingerprint of spoilage. To better elucidate the chemical basis of odor changes in fish filets during storage, the principal component analysis (PCA) of electronic nose (E-nose) responses was correlated with GC-MS-identified volatile compounds. The distinct separation observed in the PCA plot is supported by the GC-MS analysis of key spoilage volatiles. Sensors with high loadings on PC1 (W1W, W5S) primarily responded to sulfur-containing compounds and nitrogen oxides, which are typical microbial metabolites. Conversely, sensors on PC2 (W2S, W5C) detected alcohols and aldehydes, such as hexanal, consistent with lipid oxidation products. This correlation confirms that the E-nose effectively captured the specific volatile fingerprint associated with spoilage, providing a complementary validation to the chemical analysis.

#### 3.4.2. Detection of Essential Oil Compounds by Gas Chromatography-Mass Spectrometry

The composition of essential oil was analyzed Via GC-MS. A total of 55 volatile compounds were separated and identified. The compounds were confirmed by matching against the NIST mass spectral library coupled with retention index (RI) verification, and their relative contents were calculated Via the peak area normalization method (Table 2). The analysis revealed that the major constituents of the *Cinnamomum burmannii* essential oil included borneol (10.81%), 1-methyl-3-isopropylbenzene (6.40%), 4-methylene-1-isopropylbicyclo [3.1.0] hexane (5.78%), and α-terpineol (5.29%). Notably, borneol and α-terpineol are oxygenated monoterpenes that have been reported to exhibit strong bacteriostatic activity and food preservation potential [48]. The compositional breakdown revealed that total terpene hydrocarbons (monoterpene and sesquiterpene hydrocarbons) accounted for 34.23%, total oxygenated terpenes (Oxygenated mono, sesquiterpenes) accounted for 34.00%, and total nonterpenoid compounds (aldehydes, esters, etc.) constituted 31.77%. Ref. [49] further corroborated that the abundant mono- and sesquiterpenes in *Cinnamomum burmannii* essential oil serve as the primary bioactive foundation for its preservation efficacy. Although the oil composition is significantly influenced by factors such as geographical origin, harvest season, and extraction methods, its primary volatile constituents are dominated by monoterpenes, sesquiterpenes, and their oxygenated derivatives, demonstrating stable chemical characteristics and robust application potential in functional formulations.

**Figure 4:**
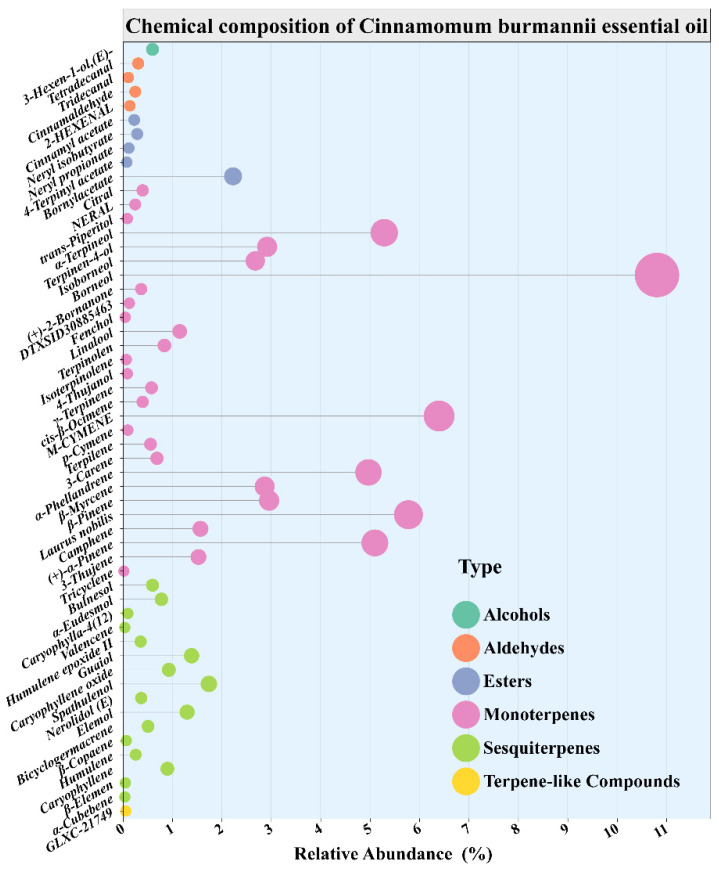
Quantitative distribution of essential oil components identified by GC-MS, visualized as a lollipop plot.

Figure 4 shows the relative quantity and chemical makeup of the chemicals in the essential oil of *Cinnamomum burmannii*, which are divided into 6 functional groups: terpene, alcohols, aldehydes, esters, monoterpenes, and sesquiterpenes. Monoterpenes (Seen by pink bubbles) predominate among these in terms of quantity and content, suggesting that the essential oil is mostly made up of tiny, volatile molecules like myrcene, limonene, and α-pinene. Interestingly, the most prevalent molecule is cinnamonaldehyde, which plays a major role in the oil’s distinctive scent and biological activity (relative area percentage of 0.25%), plays a significant role in the essential oil, especially its antibacterial and antioxidant qualities. Moderate concentrations of sesquiterpenes (in green color), which include substances like humulene oxide and caryophyllene oxide, may additionally strengthen the oil’s long-lasting pharmacological effects; the essential oil exhibits a rich and complex profile with promising applications in natural preservation, antimicrobial formulations, and fragrance products.

### 3.5. Inhibitory Activity of the Essential Oil Against Escherichia coli and Staphylococcus aureus

**Figure 5:**
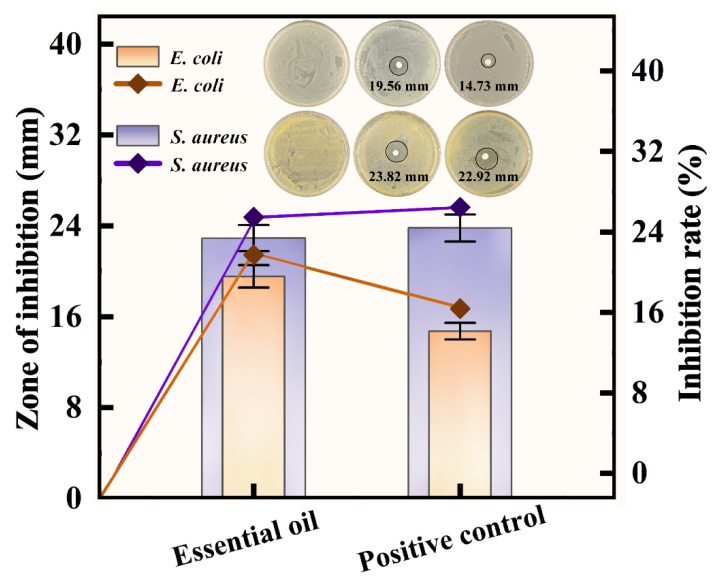
Inhibitory activity of *Cinnamomum burmannii* essential oil against *Escherichia coli* and *Staphylococcus aureus*. Left Y-axis: Inhibition zone diameter (mm); right Y-axis: Inhibition rate (%).

In this study, ampicillin was used as the positive control, while culture medium without any additives served as the blank control group [50]. As shown in Figure 5, *Cinnamomum burmannii* essential oil significantly inhibited two common foodborne pathogens. The inhibition zone diameter against *Escherichia coli* was 19.56 mm, while its activity against *Staphylococcus aureus* was more pronounced, with an inhibition zone of 22.92 mm, approaching the positive control’s efficacy (23.82 mm). The inhibitory activity of *Staphylococcus aureus* is higher than that of *Escherichia coli*, a trend that is highly consistent with the research of [40]. Due to the complex lipopolysaccharide (LPS) structure of the outer membrane of Gram-negative bacteria, they typically exhibit greater resistance to many plant extracts.

According to recent research, terpenoids increase membrane permeability by altering the LPS structure of Gram-negative bacteria’s outer membrane, which has bacteriostatic effects [51]. *Cinnamomum burmannii* essential oil’s bioactive ingredients can also block bacterial cell membrane efflux mechanisms, which stops bacteriostatic drugs from being expelled and increases their permeability and bacteriostatic activity [52]. Additionally, this is similar to the strong inhibitory activity of plant essential oils against various foodborne bacteria reported by [53], further validating the potential of plant essential oils as effective natural alternatives to combat food spoilage and pathogenic microorganisms. In conclusion, the potent bacteriostatic properties of essential oil are probably due to synergistic multitarget processes, exhibits a lesser potential for resistance induction when compared to conventional antibiotics with single-mode effects, underscoring its potential for use in bacteriostatic treatments and food preservation.

### 3.6. Preservation Efficacy of Essential Oil Preservative Films on Fish Filets and Their Color Changes During Storage

**Figure 6:**
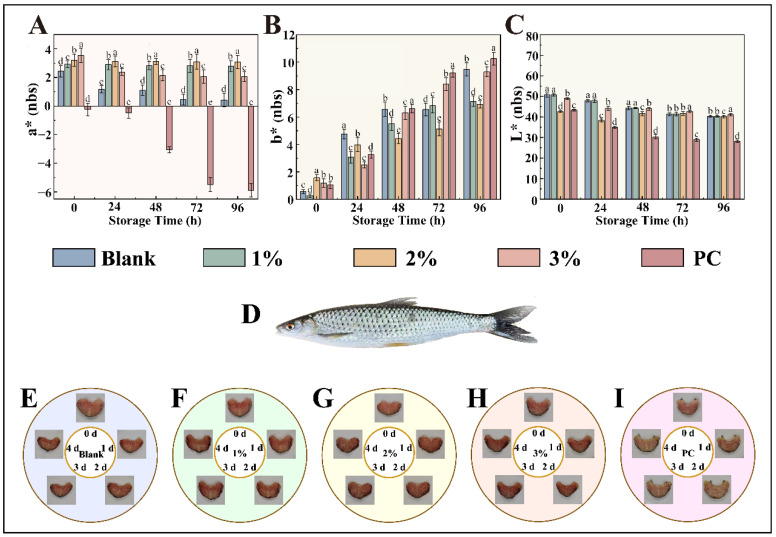
Changes in color parameters of fish filets during storage: (**A**) L* value (lightness), (**B**) a* value (redness), and (**C**) b* value (yellowness) across different treatment groups. (**D**) Whole specimen of *Squaliobarbus curriculus*. Preservation performance of fish filets treated with preservative films containing different concentrations of essential oils (**F**–**H**), blank control (**E**), and positive control (**I**). a–e: Significant differences.

As illustrated in Figure 6, the untreated fish samples (blank control group) exhibited noticeable muscle relaxation, softening, and gradual disintegration of muscle fiber structures over time. This deterioration was attributed primarily to the synergistic effects of microbial proliferation and endogenous enzymatic hydrolysis, which collectively accelerated spoilage. In contrast, fish samples treated with preservative films containing varying concentrations of essential oils maintained superior textural integrity, significantly improved quality, and prolonged shelf-life (Figure 6F–H). A positive correlation was observed between the essential oil concentration and preservation efficacy, with higher concentrations contributing to enhanced storage stability. During storage, all the samples experienced varying degrees of shrinkage, mild deformation, and progressive discoloration, with the color shifting from bright red to dark red and eventually browning. Notably, by day 4, the control group exhibited significant darkening, likely due to the oxidation of myoglobin into metmyoglobin. In contrast, samples treated with essential oil-infused films retained relatively stable coloration throughout the storage period, with the protective effect becoming more pronounced at higher oil concentrations. These results suggest that the incorporation of essential oils effectively suppressed lipid oxidation and microbial activity, thereby delaying overall quality deterioration and extending shelf-life.

The color parameters of the fish samples from the different treatment groups were quantitatively analyzed Via a chromameter (Figure 6A–C). The changes in a* values (redness) were closely linked to myoglobin oxidation. The bright red color of fresh fish is due primarily to oxymyoglobin, which is formed through the binding of oxygen to myoglobin during early postmortem stages [54]. The oxidation of myoglobin to metmyoglobin results in brownish discoloration. Fresh samples presented high a* values, indicative of low oxidation. Compared with the control samples, the essential oil-treated samples presented a smaller decrease in a* values and better redness retention after 4 days, confirming the ability of the film to inhibit myoglobin oxidation and preserve color stability.

With respect to b* values (yellowness), fresh fish initially presented low values [55]. However, the formation of yellowish compounds such as aldehydes and ketones during lipid oxidation led to a gradual increase in b* values over time. Compared with the control group, the essential oil-treated groups presented a significantly slower increase in b* values, indicating effective suppression of oxidative yellowing. Moreover, the L* values (lightness) decreased with prolonged storage, reflecting darkening [56]. However, films with higher essential oil concentrations helped maintain higher L* values, particularly in the 3% treatment group, which preserved the highest L* value after 3 days. In summary, the essential oil-enriched preservative film effectively inhibited lipid oxidation and microbial growth, thereby stabilizing key color attributes (a*, b*, and L*) and preserving the sensory quality of the fish filets throughout storage.

During storage, the changes in total bacterial colony counts of *Squaliobarbus curriculus* filets showed that the initial colony levels were similar across all groups and met fresh product standards. The blank control group exhibited rapid microbial proliferation, reaching the spoilage threshold within a short period, indicating poor preservation efficacy. In contrast, all groups treated with preservative film containing essential oils exhibited antibacterial effects, with the inhibitory effect increasing with higher essential oil concentrations. As is shown in the Table 3, the 3% treatment group demonstrated the strongest antibacterial capacity, comparable to that of the chemical preservative group. This effect is attributed to the antibacterial properties of the active components in *Cinnamomum burmannii* essential oil and the stable sustained-release effect achieved through nano-encapsulation technology, indicating its potential to replace traditional preservatives [57].

## 4. Conclusions

In this study, we developed a novel preservative film by nanoencapsulating *Cinnamomum burmannii* essential oil within zein-gum arabic nanoparticles and embedding them into a sodium alginate-gelatin matrix. Compared with the blank control, the optimized film exhibited a 32% increase in tensile strength (9.73 and 7.38 MPa), demonstrating enhanced mechanical integrity and structural stability. Biological assays confirmed that the essential oil effectively inhibited the growth of *Escherichia coli* and *Staphylococcus aureus*, highlighting its potential as a natural antimicrobial agent. Application of the film containing 3% essential oil to *Squaliobarbus curriculus* filets during refrigerated storage significantly reduced myoglobin oxidation and lipid peroxidation while preserving sensory quality, indicating superior protective performance. The high-pressure homogenization process ensured uniform nanoparticle dispersion, which not only enhanced transparency and mechanical properties but also promoted controlled release of active compounds. Overall, this work presents a functional, biodegradable, and biocompatible packaging system that outperforms conventional films by combining mechanical robustness, antimicrobial efficacy, and antioxidant capacity. The study provides a practical and sustainable strategy for high-efficiency preservation of aquatic food products, demonstrating the potential of nanoencapsulated essential oils as eco-friendly alternatives to synthetic preservatives.

The scalability of the preparation process, including high-pressure homogenization and uniform film casting, requires further investigation to ensure feasibility for industrial production. Additionally, while the preservation effects were demonstrated under controlled laboratory conditions, the potential impact on fish flavor and quality under real-world storage and distribution scenarios remains to be validated.

Looking forward, this approach can be extended by incorporating additional natural antimicrobial agents or bioactive compounds to achieve synergistic effects, further enhancing preservation efficiency and expanding the applicability of composite films for diverse food products. Such developments could offer novel solutions for sustainable and high-performance food packaging in the future.

## Figures and Tables

**Table 1:** Physical properties of preservative films containing varying concentrations of *Cinnamomum burmannii* essential oil (EO).

Sample	Film Thickness (mm)	Mean Tensile Strength (MPa)	Elongation at Break (%)	Light Transmittance (%)
Blank film	0.17 ± 0.01	6.66 ± 0.06	0.66 ± 0.01	32.28 ± 0.08
Film (1% EO)	0.20 ± 0.01	9.73 ± 0.03	1.43 ± 0.03	33.91 ± 0.11
Film (2% EO)	0.22 ± 0.01	9.15 ± 0.03	1.53 ± 0.03	34.59 ± 0.09
Film (3% EO)	0.23 ± 0.01	9.50 ± 0.05	1.37 ± 0.02	35.97 ± 0.07

**Table 2:** Gas chromatography–mass spectrometry results for the chemical composition of *Cinnamomum burmannii* essential oil.

No.	Compounds ^A^	Chemical Structure	RI ^B^	CAS	RA (%) ^C^
**1**	2-Hexenal,(E)-	C_6_H_10_O	850	006728-26-3	0.14
**2**	3-Hexen-1-ol,(E)-	C_6_H_12_O	853	000928-97-2	0.60
**3**	Tricyclo [2.2.1.0(2,6)]heptane,1,7,7-trimethyl-	C_10_H_16_	928	000508-32-7	0.02
**4**	Bicyclo [3.1.0]hex-2-ene,2-methyl-5-(1-methylethyl)-	C_10_H_16_	941	002867-05-2	1.53
**5**	(1R)-2,6,6-Trimethylbicyclo [3.1.1]hept-2-ene	C_10_H_16_	1010	007785-70-8	5.10
**6**	Camphene	C_10_H_16_	1079	000079-92-5	1.57
**7**	Bicyclo [3.1.0]hexane,4-methylene-1-(1-methylethyl)-	C_10_H_16_	1109	003387-41-5	5.78
**8**	β-Pinene	C_10_H_16_	1119	000127-91-3	2.96
**9**	β-Myrcene	C_10_H_16_	1148	000123-35-3	2.87
**10**	α-Phellandrene	C_10_H_16_	1155	000099-83-2	4.97
**11**	3-Carene	C_10_H_16_	1156	013466-78-9	0.69
**12**	1,3-Cyclohexadiene,1-methyl-4-(1-methylethyl)-	C_10_H_16_	1166	000099-86-5	0.56
**13**	p-Cymene	C_10_H_14_	1241	000099-87-6	0.10
**14**	Benzene,1-methyl-3-(1-methylethyl)-	C_10_H_14_	1244	000535-77-3	6.40
**15**	1,3,6-Octatriene,3,7-dimethyl-,(Z)-	C_10_H_16_	1245	003338-55-4	0.40
**16**	γ-Terpinene	C_10_H_16_	1248	000099-85-4	0.58
**17**	Bicyclo [3.1.0]hexan-2-ol,2-methyl-5-(1-methylethyl)-,(1α,2α,5α)-	C_10_H_18_O	1444	017699-16-0	0.09
**18**	Cyclohexene,3-methyl-6-(1-methylethylidene)-	C_10_H_16_	1447	000586-63-0	0.07
**19**	Cyclohexene,1-methyl-4-(1-methylethylidene)-	C_10_H_16_	1449	000586-62-9	0.84
**20**	Linalool	C_10_H_18_O	1498	000078-70-6	1.15
**21**	(E)-4,8-Dimethylnona-1,3,7-triene	C_11_H_18_	1499	019945-61-0	0.06
**22**	Fenchol	C_10_H_18_O	1548	001632-73-1	0.05
**23**	2-Cyclohexen-1-ol,1-methyl-4-(1-methylethyl)-,cis-	C_10_H_18_O	1593	029803-82-5	0.13
**24**	(+)-2-Bornanone	C_10_H_16_O	1598	000464-49-3	0.37
**25**	Borneol	C_10_H_18_O	1653	000507-70-0	10.81
**26**	Isoborneol	C_10_H_18_O	1659	000124-76-5	2.68
**27**	Terpinen-4-ol	C_10_H_18_O	1663	000562-74-3	2.92
**28**	α-Terpineol	C_10_H_18_O	1668	000098-55-5	5.29
**29**	2-Cyclohexen-1-ol,3-methyl-6-(1-methylethyl)-,trans-	C_10_H_18_O	1675	016721-39-4	0.09
**30**	2,6-Octadienal,3,7-dimethyl-,(Z)-	C_10_H_16_O	1676	000106-26-3	0.25
**31**	2,6-Octadienal,3,7-dimethyl-,(E)-	C_10_H_16_O	1718	000141-27-5	0.40
**32**	Cinnamaldehyde,(E)-	C_9_H_8_O	1725	014371-10-9	0.25
**33**	Bornylacetate	C_12_H_20_O_2_	1733	000076-49-3	2.23
**34**	4-Terpinenylacetate	C_12_H_20_O_2_	1738	004821-04-9	0.08
**35**	2,6-Octadien-1-ol,3,7-dimethyl-,propanoate,(Z)-	C_13_H_22_O_2_	1758	000105-91-9	0.12
**36**	α-Cubebene	C_15_H_24_	1762	017699-14-8	0.04
**37**	Propanoicacid,2-methyl-,3,7-dimethyl-2,6-octadienylester,(Z)-	C_14_H_24_O_2_	1766	002345-24-6	0.29
**38**	Cyclohexane,1-ethenyl-1-methyl-2,4-bis(1-methylethenyl)-,[1S-(1α,2β,4β)]-	C_15_H_24_	1769	000515-13-9	0.05
**39**	Tridecanal	C_13_H_26_O	1782	010486-19-8	0.11
**40**	Caryophyllene	C_15_H_24_	1788	000087-44-5	0.90
**41**	Aceticacid, cinnamylester	C_11_H_12_O_2_	1792	000103-54-8	0.23
**42**	Humulene	C_15_H_24_	1804	006753-98-6	0.26
**43**	β-Copaene	C_15_H_24_	1815	018252-44-3	0.07
**44**	Bicyclogermacrene	C_15_H_24_	1821	067650-90-2	0.51
**45**	Cyclohexanemethanol,4-ethenyl-α,α,4-trimethyl-3-(1-methylethenyl)-,[1R-(1α,3α,4β)]-	C_15_H_26_O	1866	000639-99-6	1.30
**46**	1,6,10-Dodecatrien-3-ol,3,7,11-trimethyl-,(E)-	C_15_H_26_O	1869	040716-66-3	0.37
**47**	1H-Cycloprop[e]azulen-7-ol, decahydro-1,1,7-trimethyl-4-methylene-,[1ar-(1aα,4aα,7β,7aβ,7bα)]-	C_15_H_24_O	1894	006750-60-3	1.74
**48**	Caryophylleneoxide	C_15_H_24_O	1937	001139-30-6	0.93
**49**	Guaiol	C_15_H_26_O	2065	000489-86-1	1.39
**50**	(1R,3E,7E,11R)-1,5,5,8-Tetramethyl-12-oxabicyclo [9.1.0]dodeca-3,7-diene	C_15_H_24_O	2068	019888-34-7	0.36
**51**	Tetradecanal	C_14_H_28_O	2072	000124-25-4	0.31
**52**	Naphthalene,1,2,3,5,6,7,8,8a-octahydro-1,8a-dimethyl-7-(1-methylethenyl)-,[1R-(1α,7β,8aα)]-	C_15_H_24_	2123	004630-07-3	0.04
**53**	Caryophylla-4(12),8(13)-dien-5α-ol	C_15_H_24_O	2285	019431-79-9	0.10
**54**	2-Naphthalenemethanol,1,2,3,4,4a,5,6,8a-octahydro-α,α,4a,8-tetramethyl-,[2R-(2α,4aα,8aβ)]-	C_15_H_26_O	2289	000473-16-5	0.78
**55**	5-Azulenemethanol,1,2,3,3a,4,5,6,7-octahydro-α,α,3,8-tetramethyl-,[3S-(3α,3aβ,5α)]-	C_15_H_26_O	2296	022451-73-6	0.60
	Total identified compounds				100%
	Total terpene hydrocarbons				34.23%
	Total oxygenated terpenes				34.00%
	Total non-terpenoid				31.77%

^A^ Compounds listed in order of elution from the HP-5MS capillary column; ^B^ Retention indices relative to C11–C21 n-alkanes on the HP-5MS capillary column; ^C^ Relative area percentage (peak area relative to the total peak area, %).

**Table 3:** Changes in total colony count (Log_10_ CFU/g) during storage of different *Squaliobarbus curriculus* filets.

Storage Duration (Days)	BC	1% EO	2% EO	3% EO	Positive Control
0	3.52 ± 0.08	3.56 ± 0.10	3.44 ± 0.11	3.57 ± 0.07	3.41 ± 0.17
2	7.10 ± 0.16	5.94 ± 0.64	5.83 ± 0.72	4.78 ± 0.58	5.14 ± 0.71
4	9.07 ± 0.39	7.79 ± 0.90	6.70 ± 0.73	6.75 ± 0.44	6.23 ± 0.78

BC: Blank Control; 1% EO, 2% EO, 3% EO means film with 1%, 2% and 3% essential oil.

## Data Availability

The original contributions presented in this study are included in the article. Further inquiries can be directed to the corresponding authors.

## References

[1] Tan M., Mei J., Xie J. (2021). The Formation and Control of Ice Crystal and Its Impact on the Quality of Frozen Aquatic Products: A Review. Crystals.

[2] Zheng Q., Huang F., Zheng H., Zhang H., Wen R., Li C. (2024). Chromosome-Level Genome Assembly and Annotation of Barbel Chub *Squaliobarbus curriculus*. Sci. Data.

[3] Liu M.-J., Gao J., Guo H.-Y., Zhu K.-C., Liu B.-S., Zhang N., Zhu T.-F., Zhang D.-C. (2024). Influence of Aquaculture Environments on the Muscle Quality of Golden Pompano (*Trachinotus ovatus*) in the Beibu Gulf: A Multifaceted Analysis of Nutritional, Textural, and Flavor Profiles. LWT.

[4] Daskalova A. (2019). Farmed Fish Welfare: Stress, Post-Mortem Muscle Metabolism, and Stress-Related Meat Quality Changes. Int. Aquat. Res..

[5] Erikson U., Uglem S., Greiff K. (2021). Freeze-Chilling of Whitefish: Effects of Capture, On-Board Processing, Freezing, Frozen Storage, Thawing, and Subsequent Chilled Storage—A Review. Foods.

[6] Tavares J., Martins A., Fidalgo L.G., Lima V., Amaral R.A., Pinto C.A., Silva A.M., Saraiva J.A. (2021). Fresh Fish Degradation and Advances in Preservation Using Physical Emerging Technologies. Foods.

[7] Li D., Zhu Z., Sun D.-W. (2018). Effects of Freezing on Cell Structure of Fresh Cellular Food Materials: A Review. Trends Food Sci. Technol..

[8] Liu S., Zeng X., Zhang Z., Long G., Lyu F., Cai Y., Liu J., Ding Y. (2020). Effects of Immersion Freezing on Ice Crystal Formation and the Protein Properties of Snakehead (*Channa argus*). Foods.

[9] Mahato S., Zhu Z., Sun D.-W. (2019). Glass Transitions as Affected by Food Compositions and by Conventional and Novel Freezing Technologies: A Review. Trends Food Sci. Technol..

[10] Wang Y., Miyazaki R., Saitou S., Hirasaka K., Takeshita S., Tachibana K., Taniyama S. (2018). The Effect of Ice Crystals Formations on the Flesh Quality of Frozen Horse Mackerel (*Trachurus japonicus*). J. Texture Stud..

[11] Guo C., Guo H. (2022). Progress in the Degradability of Biodegradable Film Materials for Packaging. Membranes.

[12] Huang C., Liao Y., Zou Z., Chen Y., Jin M., Zhu J., Abdalkarim S.Y.H., Zhou Y., Yu H.-Y. (2022). Novel Strategy to Interpret the Degradation Behaviors and Mechanisms of Bio- and Non-Degradable Plastics. J. Clean. Prod..

[13] Raddadi N., Fava F. (2019). Biodegradation of Oil-Based Plastics in the Environment: Existing Knowledge and Needs of Research and Innovation. Sci. Total Environ..

[14] Gheorghita R., Gutt G., Amariei S. (2020). The Use of Edible Films Based on Sodium Alginate in Meat Product Packaging: An Eco-Friendly Alternative to Conventional Plastic Materials. Coatings.

[15] Thompson R.C., Moore C.J., vom Saal F.S., Swan S.H. (2009). Plastics, the Environment and Human Health: Current Consensus and Future Trends. Philos. Trans. R. Soc. B Biol. Sci..

[16] Doğan G., İzci L. (2017). Effects on Quality Properties of Smoked Rainbow Trout (*Oncorhynchus mykiss*) Fillets of Chitosan Films Enriched with Essential Oils. J. Food Process. Preserv..

[17] Sani M.A., Azizi-Lalabadi M., Tavassoli M., Mohammadi K., McClements D.J. (2021). Recent Advances in the Development of Smart and Active Biodegradable Packaging Materials. Nanomaterials.

[18] Shaikh S., Yaqoob M., Aggarwal P. (2021). An Overview of Biodegradable Packaging in Food Industry. Curr. Res. Food Sci..

[19] Yan P., Lan W., Xie J. (2025). Characterizations of Two Physically Modified Sodium Alginate Composite Films Doped with Gallic Acid and Their Preservation of Refrigerated Sea Bass (*Lateolabrax maculatus*). Food Chem..

[20] Chen H., Zhong Q. (2015). A Novel Method of Preparing Stable Zein Nanoparticle Dispersions for Encapsulation of Peppermint Oil. Food Hydrocoll..

[21] Lisitsyn A., Semenova A., Nasonova V., Polishchuk E., Revutskaya N., Kozyrev I., Kotenkova E. (2021). Approaches in Animal Proteins and Natural Polysaccharides Application for Food Packaging: Edible Film Production and Quality Estimation. Polymers.

[22] Mohamed S.A.A., El-Sakhawy M., Nashy E.-S.H.A., Othman A.M. (2019). Novel Natural Composite Films as Packaging Materials with Enhanced Properties. Int. J. Biol. Macromol..

[23] Stoleru E., Brebu M. (2021). Stabilization Techniques of Essential Oils by Incorporation into Biodegradable Polymeric Materials for Food Packaging. Molecules.

[24] Esfanjani A.F., Jafari S.M. (2016). Biopolymer Nano-Particles and Natural Nano-Carriers for Nano-Encapsulation of Phenolic Compounds. Colloids Surf. B Biointerfaces.

[25] Kuspradini H., Putri A.S., Sukaton E., Mitsunaga T. (2016). Bioactivity of Essential Oils from Leaves of Dryobalanops Lanceolata, Cinnamomum Burmannii, Cananga Odorata, and Scorodocarpus Borneensis. Agric. Agric. Sci. Procedia.

[26] Ervina M., Lie H.S., Diva J., Caroline, Tewfik S., Tewfik I. (2019). Optimization of Water Extract of Cinnamomum Burmannii Bark to Ascertain Its in Vitro Antidiabetic and Antioxidant Activities. Biocatal. Agric. Biotechnol..

[27] Zhang S., Zhang M., Fang Z., Liu Y. (2017). Preparation and Characterization of Blended Cloves/Cinnamon Essential Oil Nanoemulsions. LWT.

[28] Dou L., Li B., Zhang K., Chu X., Hou H. (2018). Physical Properties and Antioxidant Activity of Gelatin-Sodium Alginate Edible Films with Tea Polyphenols. Int. J. Biol. Macromol..

[29] Jeyaratnam N., Nour A.H., Kanthasamy R., Nour A.H., Yuvaraj A.R., Akindoyo J.O. (2016). Essential Oil from Cinnamomum Cassia Bark through Hydrodistillation and Advanced Microwave Assisted Hydrodistillation. Ind. Crops Prod..

[30] Zhang X., Zhuang X., Chen M., Wang J., Qiu D., Liu Z., Huang Y., Zhang L., Liu Z. (2024). An Environmentally Friendly Production Method: The Pectin and Essential Oil from the Waste Peel of Juvenile Pomelo (*Citrus maxima* ‘Shatian Yu’) Were Extracted Simultaneously in One Step with an Acid-Based Deep Eutectic Solvent. LWT.

[31] Bao M., Mu H., Niu B., Liu R., Chen H., Chen H., Wang L., Han Y., Wang G., Gao H. (2025). High UV-Shielding Polyvinyl Alcohol/Chitosan-Based Transparent Bioplastic Film for Food Preservation. Food Packag. Shelf Life.

[32] Delgado-Rodríguez M., Ruiz-Montoya M., Giraldez I., López R., Madejón E., Díaz M.J. (2012). Use of Electronic Nose and GC-MS in Detection and Monitoring Some VOC. Atmos. Environ..

[33] Pennazza G., Fanali C., Santonico M., Dugo L., Cucchiarini L., Dachà M., D’Amico A., Costa R., Dugo P., Mondello L. (2013). Electronic Nose and GC–MS Analysis of Volatile Compounds in Tuber Magnatum Pico: Evaluation of Different Storage Conditions. Food Chem..

[34] Fan X., Zhang B., Zhang X., Ma Z., Feng X. (2023). Incorporating Portulaca Oleracea Extract Endows the Chitosan-Starch Film with Antioxidant Capacity for Chilled Meat Preservation. Food Chem. X.

[35] Kuttithodi A.M., Narayanankutty A., Visakh N.U., Job J.T., Pathrose B., Olatunji O.J., Alfarhan A., Ramesh V. (2023). Chemical Composition of the Cinnamomum Malabatrum Leaf Essential Oil and Analysis of Its Antioxidant, Enzyme Inhibitory and Antibacterial Activities. Antibiotics.

[36] Kaewthong P., Waiyagan K., Wattanachant S. (2017). Imaging Analysis by Digital Camera for Separating Broiler Breast Meat with Low Water-Holding Capacity. J. Poult. Sci..

[37] Baixas-Nogueras S., Bover-Cid S., Veciana-Nogués M.T., Vidal-Carou M.C. (2009). Effect of Gutting on Microbial Loads, Sensory Properties, and Volatile and Biogenic Amine Contents of European Hake (*Merluccius merluccius* Var. mediterraneus) Stored in Ice. J. Food Prot..

[38] Spanget-Larsen J., Hansen B.K.V., Hansen P.E. (2011). OH Stretching Frequencies in Systems with Intramolecular Hydrogen Bonds: Harmonic and Anharmonic Analyses. Chem. Phys..

[39] Liu Y., Zhao A., Zheng Y., Zhu X., Hu Y., Qu X. (2024). Scented Solutions: Harnessing Lavender Essential Oil Liposomes for Enhanced Plywood Performance. Sustain. Chem. Pharm..

[40] Motelica L., Ficai D., Petrisor G., Oprea O.-C., Trușcǎ R.-D., Ficai A., Andronescu E., Hudita A., Holban A.M. (2024). Antimicrobial Hydroxyethyl-Cellulose-Based Composite Films with Zinc Oxide and Mesoporous Silica Loaded with Cinnamon Essential Oil. Pharmaceutics.

[41] Munhuweyi K., Caleb O.J., Lennox C.L., van Reenen A.J., Opara U.L. (2017). In Vitro and in Vivo Antifungal Activity of Chitosan-Essential Oils against Pomegranate Fruit Pathogens. Postharvest Biol. Technol..

[42] Liang Q., Cao P., Lu H., Du Y., Kang L., Ma H., Ren X. (2025). Ultrasonic Engineering of Zein-Curcumin Nanoparticles/Sodium Alginate Composite Films for Active Food Packaging Applications. Int. J. Biol. Macromol..

[43] Rui L., Li Y., Wu X., Wang Y., Xia X. (2024). Effect of Clove Essential Oil Nanoemulsion on Physicochemical and Antioxidant Properties of Chitosan Film. Int. J. Biol. Macromol..

[44] Rodriguez-Caturla M.Y., Margalho L.P., Graça J.S., Pia A.K.R., Xavier V.L., Noronha M.F., Cabral L., Lemos-Junior W.J.F., Castillo C.J.C., Sant’Ana A.S. (2025). Bacterial Dynamics and Volatile Metabolome Changes of Vacuum-Packaged Beef with Different PH during Chilled Storage. Int. J. Food Microbiol..

[45] Velázquez-Contreras F., Zamora-Ledezma C., López-González I., Meseguer-Olmo L., Núñez-Delicado E., Gabaldón J.A. (2022). Cyclodextrins in Polymer-Based Active Food Packaging: A Fresh Look at Nontoxic, Biodegradable, and Sustainable Technology Trends. Polymers.

[46] Martin D., Joly C., Dupas-Farrugia C., Adt I., Oulahal N., Degraeve P. (2023). Volatilome Analysis and Evolution in the Headspace of Packed Refrigerated Fish. Foods.

[47] Qu Y., Yun J., Li Y., Ai D., Zhang W. (2023). Microbial Succession and Its Correlation with the Dynamics of Flavor Compounds Involved in the Fermentation of Longxi Bacon. Front. Microbiol..

[48] Skočibušić M., Bezić N. (2004). Phytochemical Analysis and in Vitro Antimicrobial Activity of Two Satureja Species Essential Oils. Phyther. Res..

[49] Budiastuti, Andini Y.W., Cahyasarl I.A., Primaharinastiti R., Sukardiman (2020). Standardization Bark of Cinnamomum Burmannii Nees Ex Bl. from Five Areas of Indonesia. Pharmacogn. J..

[50] Chairunnisa, Tamhid H.A., Nugraha A.T. (2017). Gas Chromatography—Mass Spectrometry Analysis and Antibacterial Activity of Cinnamomum Burmanii Essential Oil to Staphylococcus Aureus and Escherichia Coli by Gaseous Contact. AIP Conf. Proc..

[51] Chen X., Liu P., Wang J., He X., Wang J., Chen H., Wang G. (2024). TMT-Based Quantitative Proteomics Revealed the Antibacterial Mechanism of Cinnamaldehyde against MRSA. J. Proteome Res..

[52] Rachmawati A., Thohari I., Al Awwaly K.U., Apriliyani M.W. (2023). Antimicrobial Activity of Edible Film with Cinnamon Essential Oil as Antimicrobial Packaging. J. Ilmu dan Teknol. Has. Ternak.

[53] Maniki E., Kostoglou D., Paterakis N., Nikolaou A., Kourkoutas Y., Papachristoforou A., Giaouris E. (2023). Chemical Composition, Antioxidant, and Antibiofilm Properties of Essential Oil from *Thymus capitatus* Plants Organically Cultured on the Greek Island of Lemnos. Molecules.

[54] Thiansilakul Y., Benjakul S., Richards M.P. (2011). The Effect of Different Atmospheric Conditions on the Changes in Myoglobin and Colour of Refrigerated Eastern Little Tuna (*Euthynnus affinis*) Muscle. J. Sci. Food Agric..

[55] Erdağ M., Ayvaz Z. (2021). The Use of Color to Determine Fish Freshness: European Seabass (*Dicentrarchus labrax*). J. Aquat. Food Prod. Technol..

[56] Karamucki T., Jakubowska M., Rybarczyk A., Gardzielewska J. (2013). The Influence of Myoglobin on the Colour of Minced Pork Loin. Meat Sci..

[57] Zhong H., Wei S., Kang M., Sun Q., Xia Q., Wang Z., Han Z., Liu Y., Liu M., Liu S. (2023). Effects of Different Storage Conditions on Microbial Community and Quality Changes of Greater Amberjack (*Seriola dumerili*) Fillets. LWT.

